# QTL identification and KASP marker development for productive tiller and fertile spikelet numbers in two high-yielding hard white spring wheat cultivars

**DOI:** 10.1007/s11032-018-0894-y

**Published:** 2018-11-01

**Authors:** Rui Wang, Yuxiu Liu, Kyle Isham, Weidong Zhao, Justin Wheeler, Natalie Klassen, Yingang Hu, J. Michael Bonman, Jianli Chen

**Affiliations:** 1 Department of Plant Sciences, University of Idaho, Aberdeen, ID USA; 2 State Key Laboratory of Crop Stress Biology for Arid Areas, College of Agronomy, Northwest A&F University, Yangling, Shanxi China; 3 Small Grains and Potato Germplasm Research Unit, USDA-ARS, Aberdeen, ID USA

**Keywords:** *Triticum aestivum* L., Productive tiller, Fertile spikelet, QTL, SNP

## Abstract

Selecting high-yielding wheat cultivars with more productive tillers per unit area (PTN) combined with more fertile spikelets per spike (fSNS) is difficult. QTL mapping of these traits may aid understanding of this bottleneck and accelerate precision breeding for high yield via marker-assisted selection. PTN and fSNS were assessed in four to five trials from 2015 to 2017 in a doubled haploid population derived from two high-yielding cultivars “UI Platinum” and “SY Capstone.” Two QTL for PTN (*QPTN.uia-4A* and *QPTN.uia-6A*) and four QTL for fSNS (*QfSNS.uia-4A*, *QfSNS.uia-5A*, *QfSNS.uia-6A*, and *QfSNS.uia-7A*) were identified. The effects of the QTL were primarily additive and, therefore, pyramiding of multiple QTL may increase PTN and fSNS. However, the two QTL for PTN were positioned in the flanking regions for the two QTL for fSNS on chromosomes 4A and 6A, respectively, suggesting either possible pleiotropic effect of the same QTL or tightly linked QTL and explaining the difficulty of selecting both high PTN and fSNS in phenotypic selection. Kompetitive allele-specific PCR (KASP) markers for all identified QTL were developed and validated in a recombinant inbred line (RIL) population derived from the same two cultivars. In addition, KASP markers for three of the QTL (*QPTN.uia-6A*, *QfSNS.uia-6A*, and *QfSNS.uia-7A*) were further validated in a diverse spring wheat panel, indicating their usefulness under different genetic backgrounds. These KASP markers could be used by wheat breeders to select high PTN and fSNS.

**Electronic supplementary material:**

The online version of this article (10.1007/s11032-018-0894-y) contains supplementary material, which is available to authorized users.

## Introduction

Wheat (*Triticum aestivum* L.) is one of the most important crops globally and is a key source of carbohydrates and vegetable protein for human food consumption (Asseng et al. 2011). Improving wheat grain yield is crucial to meet the food requirements of an increasing human population. Selecting for increased grain yield generally results in changes to three yield components: productive spike (tiller) number per unit area (PTN), kernel weight (KW), and kernel number per spike (KNS), which are determined by kernel number per spikelet and fertile spikelet number per spike (fSNS). For many sink-limited wheat lines, increasing PTN and/or fSNS is a common approach to increase grain yield because of the limited potential for increasing KW and relative low heritability of the KNS compared to fSNS (Zhang et al. 2018).

QTL mapping of the PTN and fSNS produced inconsistent results with respect to QTL number, chromosomal locations, and the effects. With the advent of advanced genotyping platforms (SSR, DArT, SNP, GBS), QTL associated with the PTN have been mapped on 1D, 2D, and 6A (Li et al. 2002); 3A (Kuraparthy et al. 2007); 6B (Naruoka et al. 2011); 2D (*Qltn.sicau*-*2D*), 2B (*Qltn.sicau*-*2B*), and 5A (*Qltn.sicau*-*5A*) (Wang et al. 2016); and 4D (*QSR.sicau-4D*) (Hu et al. 2017). For fSNS, Ma et al. (2007) detected seven QTL on chromosomes 1A, 2D, 3B, 6A, 7A, and 7D, which had dominant and epistatic effects. Cui et al. (2012) detected three QTL on chromosomes 2A, 5A, and 7B for fSNS that were significant across multiple environments in two recombinant inbred line (RIL) populations. Recently, Zhai et al. (2016) identified four genomic regions affecting fSNS on chromosomes 1A, 1B, 3A, and 7A. Zhou et al. (2017) detected one QTL for fSNS on chromosome 1A in multiple environments using a doubled haploid (DH) soft red winter wheat population. Liu et al. (2018a) identified a QTL (*QFSN4B.4-17*) responsible for fSNS in multiple environments using a RIL population of 173 lines derived from a cross of the common winter wheat lines Shannong 01-35 and Gaocheng 9411.

Although many QTL for PTN and fSNS have been reported, the number of consistent QTL is limited. The major reason for this lack of consistency may be the type of the mapping populations used. Most of populations were derived from crosses of elite by unadapted parents, and the phenotypic assessment was strongly affected by the environments and by the segregation of major genes controlling plant height (*RHT1* and/or *RHT2*), photoperiod response (*PPD*), and vernalization (*VRN*). In the present study, we used a DH population that was derived from two well-adapted cultivars, UI Platinum (UIP) and SY Capstone (SYC), which have the same *RHT* and *PPD* genes. The two parents have contrasting PTN and observable difference in fSNS. The QTL analysis was conducted with high-resolution linkage map to identify QTL responsible for the observed phenotypic variation for PTN and fSNS. Breeder-friendly Kompetitive allele-specific PCR (KASP) markers were developed based on the markers associated with the QTL and were validated in F_5_-derived recombinant lines developed from the two parental cultivars.

## Materials and methods

### Mapping population and field experiments

A mapping population of 110 DH lines was developed from the cross between the two high-yielding wheat cultivars, UIP and SYC. UIP was developed and released by the Idaho Agricultural Experiment Station in 2014 (Chen et al. 2016). SYC was developed and released by Syngenta Cereals in 2011. A set of 600 F_5_ RILs was developed from the same parents and used in QTL validation. In addition, a diverse spring wheat panel was used for QTL validation. The panel was described in Wang et al. (2017). Briefly, it consists 167 cultivars or elite lines from four programs in PNW area of the USA (University of Idaho, University of California, Washington State University, Montana State University), and International Maize and Wheat Improvement Center (CIMMYT). The whole or part of the panel has been used for genome-wide association study or genomic selection study of Fusarium head blight (Wang et al. 2017; Dong et al. 2018), grain yield and plant water status (Zhang et al. 2018), and agronomic traits (Godoy et al. 2018), as parts of the Triticeae Coordinated Agricultural Project (TCAP, https://www.triticeaecap.org/).

Field experiments for the DH population were performed in five trials at Aberdeen, ID (42.96° N, 112.83° W, elevation 1342 m), during the 2015 to 2017 cropping seasons. For these trials, the DH population and parents were planted in randomized complete blocks with two replicates. The plot was 3 m long and 1.5 m wide with seven rows. Sowing density was adjusted by 1000 kernel weight and at 0.48 million seeds per hectare for each trial. The 600 F_5_ RILs were space planted with 20 seeds per row, 2.5 cm apart in the field at Aberdeen in 2017. The diverse spring wheat panel was planted with standard yield plots at Aberdeen, ID, in 2017 to 2018 and at Walla Walla, WA, in 2018. For optimal trial management, standard fertilizer application and weed control were done and wheat borders were planted to minimize edge effect.

### Trait evaluation and data analysis

For the DH population, fSNS data were collected in all five trials and PTN data were collected in four of the five trials. For the diverse spring wheat panel, the fSNS was collected in the two trials at Aberdeen in 2017 and 2018 while the PTN was collected in the trial at Aberdeen in 2017 and at Walla Walla in 2018. For the above two populations, fSNS was assessed from ten spikes randomly sampled from the fourth row of each plot and the average was calculated. The PTN was assessed before harvesting as the number of productive tillers per 45 cm in the fourth row of each plot, then converted to tiller number per square meter. For the 600 RILs, fSNS was assessed from ten randomly selected spikes in each row and PTN was assessed as the number of productive tillers per plant in ten single plants in the middle of each row.

Based on the number of trials, five datasets for fSNS (15AB_1, 15AB_2, 16AB, 17AB_1, and 17AB_2) and four for PTN (15AB_1, 15AB_2, 16AB, and 17AB_1) were created for the DH population. The best linear unbiased predictions (BLUPs) across different trials for each trait were calculated in R using package “lme4” (Bates et al. 2015; R Core Team 2016). The genotypes, trials, and replications were all considered random effects in the model. For each trait, the single-trial phenotypic datasets and the BLUP dataset were used for further analysis.

Broad-sense heritability (*H*^2^) was estimated based on the equations ${H}^2={\sigma}_{\mathrm{G}}^2/\left({\sigma}_{\mathrm{G}}^2+{\sigma}_{\mathrm{G}\mathrm{E}}^2/e+{\sigma}_e^2/ re\right)$ and ${H}^2={\sigma}_{\mathrm{G}}^2/\left({\sigma}_{\mathrm{G}}^2+{\sigma}_{\mathrm{G}\mathrm{E}}^2/\mathrm{r}+{\sigma}_{\mathrm{e}}^2/ re\right),$ as described in Wang et al. (2017). The phenotype distributions were analyzed and illustrated using histogram plots in JMP Genomics statistical suite v8.0. All distributions were fitted with normal curves in JMP Genomics v8.0 and tested with the Shapiro-Wilk/Kolmogorov method to infer normality. Furthermore, correlation coefficients among different trials and among different traits were calculated in JMP Genomics v8.0 using the default statistic method.

### Genotypic data and linkage map construction

The DH lines and two parents were genotyped with the 90K SNP iSelect platform (Wang et al. 2014) at the USDA-ARS Small Grains Genotyping laboratory in Fargo, ND. SNP were called in GenomeStudio 2011.1 using the Polyploid Clustering Module V1.0 (Illumina, San Diego, CA). In addition, 300 SSR markers were selected to genotype the DH population and parents according to the protocol described in Chen et al. (2012) as well as six STS (sequence tag site) markers for genes controlling photoperiod (*PPD-D1*), vernalization (*VRN-A1*, *VRN-B1*, *and VRN-D1*), and plant height (*RHT-B1* and *RHT-D1*).

All genotyped markers were filtered by excluding those either monomorphic or with high frequencies of missing values (≥ 10%). The segregation ratio for each marker was tested using chi-square goodness of fit and the *Q* value (FDR adjusted *P* value) at 0.0001 was used as a cutoff for excluding markers showing distorted segregation. The number of marker groups was determined with the automated hierarchical clustering method in JMP Genomics 8.0. The markers in each cluster were ordered using the Kosambi mapping function and the accelerated map order optimization algorithm in JMP Genomics 8.0. Groups were broken into parts if the genetic distance between adjacent markers was greater than 35 cM (Liu et al. 2018b).

### QTL analyses

QTL analyses to identify major or minor QTL for the two traits were performed using the composite interval mapping (CIM) model in JMP Genomics 8.0 with all single datasets and the BLUP datasets. The forward regression method was used with a window size of 10.0 cM, control marker number at 5, and a test step of 2 cM. The expectation maximization (EM) algorithm at a threshold of 2.5 (LOD > 2.5) was used to identify a significant QTL. The proportion of phenotypic variance (*R*^2^) and the additive effects of the QTL were obtained from the software output. The effect contribution from SYC or UIP was indicated by negative and positive numbers of the additive effect, respectively.

Multiple interval mapping (MIM) in JMP Genomics was conducted using BLUP data to estimate the QTL × QTL interaction effects between different QTL. These effects were estimated by Haley-Knott regression algorithm with a test window size at 10 cM. LOD at 2.5 was set as the threshold for entry and staying in the MIM model.

To characterize the physical positions of the identified QTL, QTL-associated SNP marker sequences were aligned with respect to the newly released Chinese Spring sequence (Reference Sequence v1.0, the International Wheat Genome Consortium (IWGSC), http://wheat-urgi.versailles.inra.fr/) through a BLAST search.

### KASP assays and QTL validation using selected RILs and a diverse spring wheat panel

SNP markers highly associated with a specific QTL were selected and converted to KASP markers using PolyMarker (Ramirez-Gonzalez et al. 2015). KASP assays were performed in a CFX96 Touch™ real-time PCR detection system (Bio-Rad, Hercules, CA). The reaction system and PCR conditions were based on the protocol from LGC Genomics. The plate was read at 25 °C at the last step and the data were visualized and analyzed using allelic discrimination function in CFX Maestro software (Bio-Rad, Hercules, CA).

Only the KASP markers showing the same segregation as the corresponding SNP markers in parents and the DH population were used to screen the two validation populations. Homozygous RILs were selected based on a combination of KASP marker alleles for a specific QTL. *t* tests were conducted to compare the two alleles’ effect on PTN and fSNS using selected RILs and the diverse spring wheat panel.

## Results

### Phenotypic analysis for PTN and fSNS

**Fig. 1:**
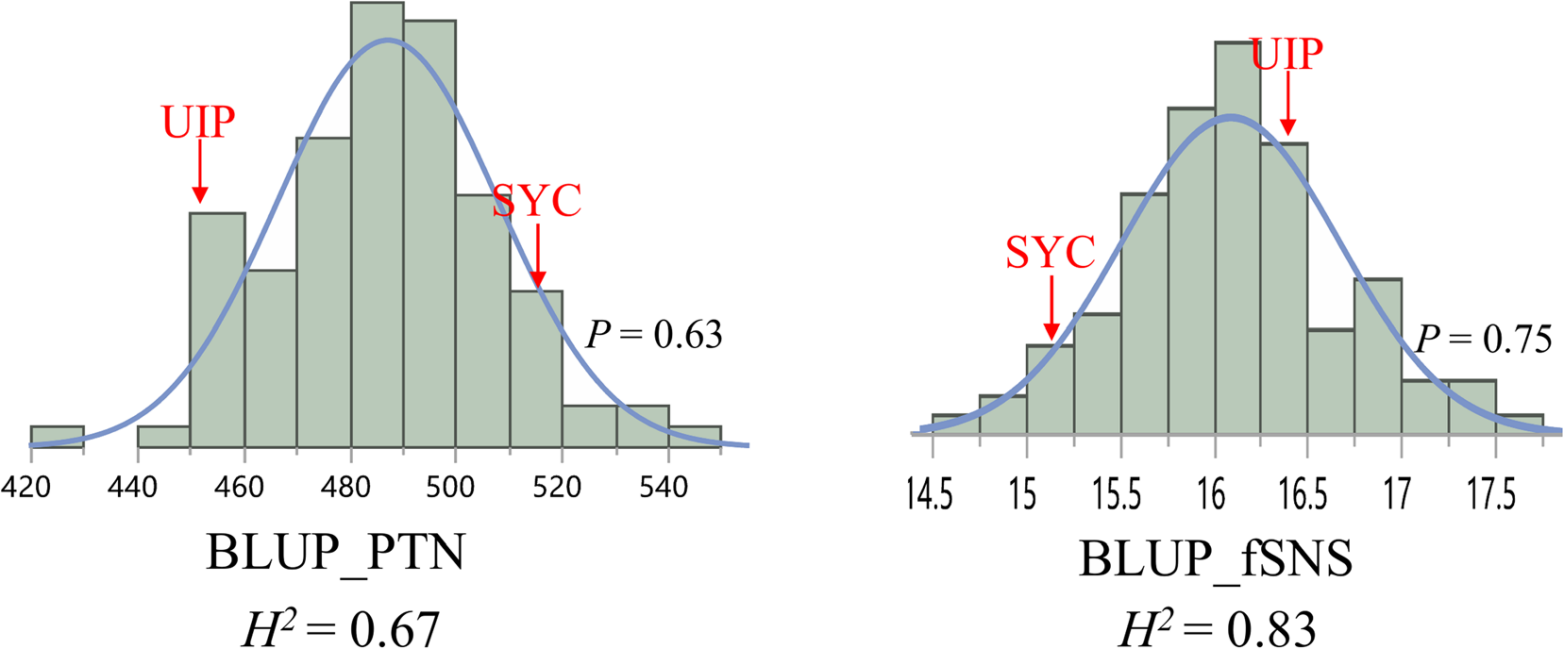
Distribution for the BLUP data of PTN and fSNS in the UIP × SYC DH population. The BLUP values for two parents were indicated on the histogram plots using red arrows. The broad-sense heritability (*H*^2^) for each trait was shown under each histogram plot

BLUP datasets for both PTN and fSNS showed normal distributions with *P* values at 0.63 and 0.75, suggesting the polygenic inheritance of these traits (Fig. 1). The parent UIP had more fSNS whereas the parent SYC had more PTN (Fig. 1). Transgressive segregation was observed for the two traits, suggesting the two parents contain different genes for the traits investigated (Fig. 1).

The PTN and fSNS showed high broad-sense heritability at 0.67 and 0.83, respectively, based on four or five trials in 3 years (Fig. 1), indicating adequate levels of genetic effect for these traits in the population. Moderate to high correlations were observed for PTN among different trials (Table 1). The PTN values from the three environments in 2015 and 2016 were strongly correlated (*r*^2^ ranged from 0.47 to 0.82), while the PTN values in 2017 (17AB_1) were moderately correlated (*r*^2^ ranged from 0.29 to 0.36) with the three environments in 2015 and 2016. Similar to the PTN results, the fSNS in all environments showed strong correlations with each other except for the environment 17AB_1, which showed a moderately high correlation with the other environments (*r*^2^ ranged from 0.37 to 0.45) (Table 1). The BLUP dataset for each trait was significantly (*P* < 0.0001) associated with all individual trials (*r*^2^ ranged from 0.62 to 0.86) (Table 1) and therefore was used in the further data analysis.

**Table 1:** Correlations among different environments for PTN and spikelet component traits

PTN	15AB_1	15AB_2	16AB	17AB_1	
15AB_2	0.82***				
16AB	0.47***	0.49***			
17AB_1	0.36**	0.30*	0.29*		
BLUP_PTN	0.77***	0.78***	0.71***	0.68***	
fSNS	15AB_1	15AB_2	16AB	17AB_1	17AB_2
15AB_2	0.88***				
16AB	0.44***	0.42***			
17AB_1	0.38***	0.37**	0.45***		
17AB_2	0.46***	0.46***	0.57***	0.66***	
BLUP_fSNS	0.86***	0.86***	0.72***	0.62***	0.78***
	BLUP_PTN				
BLUP_fSNS	− 0.44***				

Significance level: ***P < 0.0001, **P < 0.01, *P < 0.05. *ns* not significant

Correlation analysis using BLUP data showed that PTN had a strong negative correlation with fSNS at − 0.44 (*P* < 0.0001) (Table 1). This negative correlation was also found in all single dataset with *P* < 0.01 (data not shown).

### Marker analysis and linkage group construction

Of the 81,587 SNP markers on the wheat 90K SNP iSelect platform and 300 SSR markers, 9944 SNP markers and 46 SSR markers were polymorphic between the parents and among the DH population. For the six STS markers of phenology-related genes, only the three vernalization genes were polymorphic for the parents and among the DH population.

By combining all polymorphic markers and excluding markers missing in more than 10% of the lines, 1086 marker loci that were not co-located were selected for the genetic map construction. Forty-three linkage groups (LGs) were identified, which represented all 21 wheat chromosomes. Total marker density was an average of one marker each 0.37 cM and varied among chromosomes, from 0.19 (6A) to 5.56 (3D). The total length of A, B, and D genomes was 1420.51 cM, 1571.15 cM, and 641.53 cM, respectively, with average distance between markers of 0.33 cM, 0.36 cM, and 0.61 cM, respectively. The D genome had the lowest marker coverage, representing the low polymorphism in this genome.

### QTL detection for PTN and fSNS

**Fig. 2:**
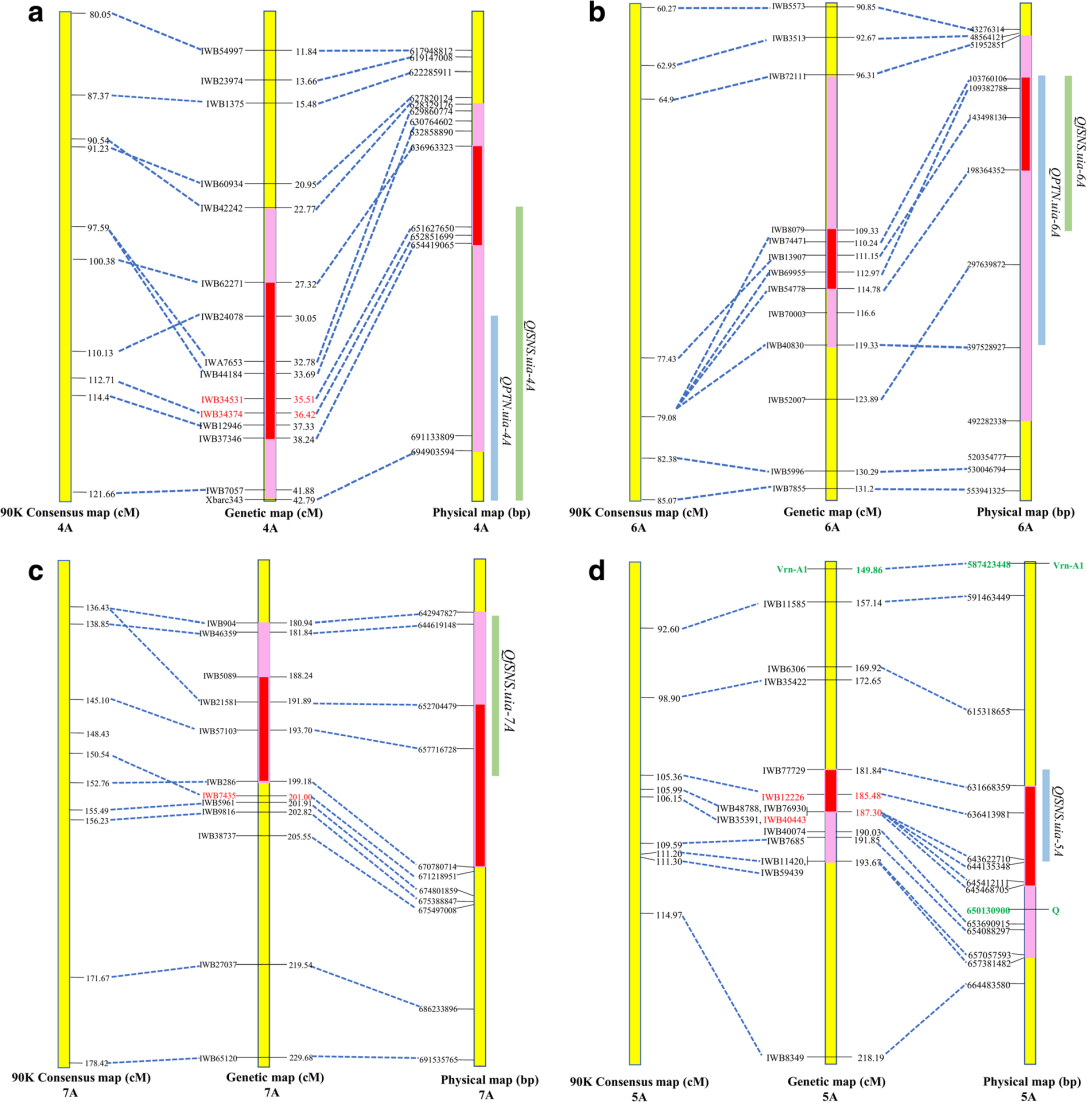
Physical positions for four QTL/QTL pairs on chromosomes 4A (**a**), 6A (**b**), 7A (**c**), and 5A (**d**). Collinearity relationships among the genetic map from the present study and the 90K consensus map and the physical map for the identified QTL/QTL pairs were indicated by dash lines on the corresponding chromosomes. The markers highlighted in red were used for KASP marker development. Pink bars on the chromosomes indicate the positions of the QTL/QTL pair flanking regions and the red bars indicate the peak regions. All QTL were indicated by green (high number allele from UIP) or blue (high number allele from SYC) bars based on the genetic positions detected in the BLUP datasets

Two QTL on chromosomes 4A and 6A were identified for PTN (Table 2 and Fig. 2a, b). *QPTN.uia-4A* was detected in BLUP_PTN, 15AB_1, and 15AB_2 and explained 15 to 19% of phenotypic variation, which represents 16~23 productive tillers per square meter in different trials (Table 2). *QPTN.uia-6A* was detected in the BLUP data and three of four environments (15AB_1, 15AB_2, and 16AB). This QTL explained 15 to 26% of phenotypic variation, which represents 22~34 productive tillers per square meter in different trials (Table 2). The high tiller number allele for both QTL was contributed by SYC.

**Table 2:** Significant QTL for PTN and spikelet component traits identified in different environments and in the BLUP dataset

Trait	QTL	Environment	Interval	Positions	Peak marker	Peak position (cM)	LOD	Effect^a^	*R*^2^ (%)
PTN	*QPTN.uia-4A*	15AB_1	*IWB1375–IWB7349*	19.48–36.42	*IWB62271*	27.32	4.05	− 23.05	19
		15AB_2	*IWB24078–IWB7057*	32.05–41.88	*IWB51174*	41.88	3.93	− 20.74	15
		BLUP_PTN	*IWB24078–Xbarc343*	30.05–48.79	*IWB37346*	38.24	3.83	− 16.20	15
	*QPTN.uia-6A*	15AB_1	*IWB72111–IWB40830*	100.31–123.33	*IWB13907*	111.15	5.13	− 25.98	19
		15AB_2	*IWB72111–IWB40830*	104.31–123.33	*IWB13907*	111.15	4.32	− 21.98	17
		16AB	*IWB72111–IWB40830*	96.31–123.33	*IWB69955*	112.97	3.79	− 33.62	15
		BLUP_PTN	*IWB72111–IWB40830*	96.31–123.33	*IWB6351*	110.24	6.74	− 23.95	26
fSNS	*QfSNS.uia-4A*	16AB	*IWB42242–Xbarc343*	22.77–44.79	*IWB34531*	33.69	5.22	0.70	20
		17AB_1	*IWB42242–Xbarc343*	22.77–48.79	*IWB34531*	35.51	9.39	0.75	32
		17AB_2	*IWB42242–Xbarc343*	22.77–48.79	*IWB34531*	35.51	9.18	0.75	32
		BLUP_fSNS	*IWB42242–Xbarc343*	22.77–48.79	*IWB34531*	35.51	11.63	0.54	39
	*QfSNS.uia-5A*	15AB_1	*IWB77729–IWB11420*	181.84–193.67	*IWB12226*	185.48	10.05	− 0.95	38
		15AB_2	*IWB77729–IWB11420*	181.84–193.67	*IWB12226*	185.48	9.10	− 0.83	35
		16AB	*IWB77729–IWB11420*	181.84–193.67	*IWB12226*	185.48	4.38	− 0.66	17
		17AB_1	*IWB35422–IWB7685*	180.65–191.85	*IWB77729*	181.84	6.24	− 0.61	23
		17AB_2	*IWB77729–IWB11420*	181.84–193.67	*IWB12226*	185.48	5.44	− 0.57	20
		BLUP_fSNS	*IWB77729–IWB11420*	181.84–193.67	*IWB12226*	185.48	8.65	− 0.45	30
	*QfSNS.uia-6A*	16AB	*IWB72111–IWB40830*	96.31–123.33	*IWB69955*	112.97	5.00	0.68	19
		17AB_1	*IWB52007–IWB52007*	127.89–129.89	*IWB52007*	127.89	2.55	0.49	10
		17AB_2	*IWB72111–IWB40830*	104.31–121.33	*IWB40830*	119.33	3.56	0.47	14
		BLUP_fSNS	*IWB72111–IWB72111*	96.31–108.31	*IWB7541*	102.31	4.12	0.33	16
	*QfSNS.uia-7A*	15AB_1	*IWB5089–IWB5961*	188.24–201.91	*IWB21581*	191.89	3.01	0.65	13
		15AB_2	*IWB5089–IWB286*	188.24–199.18	*IWB21581*	191.89	2.74	0.62	11
		17AB_1	*IWB286–IWB38737*	199.18–205.55	*IWB5961*	201.91	2.95	0.40	12
		17AB_2	*IWB5089–IWB5961*	188.24–201.91	*IWB21581*	191.89	6.03	0.79	23
		BLUP_fSNS	*IWB904–IWB286*	188.24–199.18	*IWB5089*	188.24	4.96	0.33	19

^a^The effect contribution from SYC or UIP was indicated by negative or positive number, respectively

A total of four QTL were detected for fSNS (Fig. 2a–d) on chromosomes 4A, 5A, 6A, and 7A. Among the four QTL, the high number allele of *QfSNS.uia-4A*, *QfSNS.uia-6A*, and *QfSNS.uia-7A* was contributed by UIP, while the high number allele of *QfSNS.uia-5A* was contributed by SYC. The QTL *QfSNS.uia-4A* had the largest effect of increasing up to 0.75 fertile spikelet per spike and explained 20 to 39% of phenotypic variation in three of five trials and the BLUP dataset (Table 2).

Based on the QTL interval and peak marker positions, *QfSNS.uia-4A* and *QfSNS.uia-6A* were mapped to the flanking regions of the QTL identified for PTN (Fig. 2a, b, and Table 2), suggesting these two regions contain either a single QTL with pleiotropic effects or more than one tightly linked QTL affecting both fSNS and PTN.

### Trade-off effect of single QTL pair on two traits

For QTL pairs on chromosomes 4A and 6A, the high number allele of *QPTN.uia-4A* and *QPTN.uia-6A* was contributed by SYC, whereas the high number allele of *QfSNS.uia-4A* and *QfSNS.uia-6A* was contributed by UIP, leading to a trade-off effect between PTN and fSNS for these two QTL pairs, which is supported by the allele analysis. As shown in Table 3, the lines with UIP alleles for the QTL pairs showed fewer PTN, but more fSNS than those with SYC alleles.

**Table 3:** Allele effect and the trade-off effect of single QTL/QTL pair

QTL/QTL pair^a^	Trait	Mean	SD	Pooled SD	Diff. mean	Diff. in SD	*P* value	Sample size	Power (1 − *β*)^b^
QTL-4A									
UIP alleles^c^	PTN	479.94^d^	21.69	19.67	13.58	0.69	0.0005	52/58	0.94
SYC alleles		493.52	17.65						
UIP alleles	fSNS	16.38	0.49	0.50	0.59	1.17	< 0.0001	55/55	1.00
SYC alleles		15.79	0.52						
QTL-5A									
UIP allele	fSNS	15.82	0.47	0.52	0.51	0.97	< 0.0001	51/59	1.00
SYC allele		16.32	0.57						
QTL-6A									
UIP alleles	PTN	478.63	19.68	19.08	16.34	0.86	< 0.0001	53/57	0.99
SYC alleles		494.97	18.49						
UIP alleles	fSNS	16.31	0.59	0.54	0.43	0.79	< 0.0001	54/56	0.98
SYC alleles		15.88	0.50						
QTL-7A									
UIP alleles	fSNS	16.28	0.57	0.56	0.33	0.59	0.003	46/64	0.80
SYC alleles		15.95	0.56						

^a^QTL-4A, QTL-5A, QTL-6A, and QTL-7A stand for the four QTL/QTL pairs on the four chromosomes

^b^The detection power (1 − *β*) was calculated using the online tool at https://www.stat.ubc.ca/~rollin/stats/ssize/n2.html with *α* (type I error rate) at 0.05

^c^UIP or SYC allele group stands for the lines with the alleles of all associated markers for a single QTL or multiple QTL in a specific QTL pair come from UIP or SYC

^d^The BLUP data for each trait was used to estimate the allele effect of each QTL and the statistical detection power. *t* test analyses were used to compare the two different allele groups

### Additive effect among different QTL within each trait

To better understand the relationship among the QTL identified for PTN and fSNS, QTL × QTL interactions were analyzed using MIM in JMP Genomics. The results showed that the QTL on chromosomes 4A and 6A for PTN and those on chromosomes 4A, 5A, 6A, and 7A for fSNS are additive (*P* < 0.0001) towards increasing PTN and fSNS and there was no interaction (epistasis) among them (*P* > 0.05) (Supplemental Table 1). The allelic effect analyses also supported this finding. The lines with all positive alleles showed 30.81 more PTN and 1.74 more fSNS than those without any of the positive alleles (Supplemental Fig. 1).

### Validation of the QTL effects using KASP markers in the selected RILs

For the QTL on chromosomes 4A, 5A, 6A, and 7A, two, three, and one QTL-associated SNP markers were converted to KASP markers, respectively, representing the peak or flanking markers for all identified QTL on these chromosomes (Supplemental Table 2). Based on the genotyping data of the eight markers for the 600 F_5_ RILs, the homozygous lines of each parent allele for each QTL/QTL pair were selected for further validation. fSNS was measured in all 600 RILs while the PTN was measured for 224 lines selected on the basis of their genotype for the two QTL pairs on chromosomes 4A and 6A. For each QTL/QTL pair, the QTL effects on PTN and/or fSNS were consistently detected as in the DH population. Most *P* values from the comparisons were less than 0.0001 and one was less than 0.001. The *P* values from the comparisons for *QfSNS.uia-7A* showed less significance (< 0.01) (Supplemental Table 3), possibly due to the greater distance between the developed KASP marker and the peak markers for this QTL compared to the other QTL (Fig. 2c). In addition, the trade-off effect of QTL pairs on chromosomes 4A and 6A for multiple traits and the additive effects among different QTL on all four chromosomes within each trait were also validated using the RIL population (data not shown).

### Validation of the QTL effects in the diverse spring wheat panel

All eight KASP markers were successfully genotyped in the diverse spring wheat panel and the allelic analyses were conducted based on 2 years’ phenotyping data. For fSNS, the positive alleles of *QfSNS.uia-7A* and *QfSNS.uia-6A* showed significant (*P* < 0.001 and 0.01, respectively) more fSNS at 0.83 and 0.62, respectively, than the negative alleles. The positive alleles of *QfSNS.uia-5A* and *QfSNS.uia-4A* increased 0.13 to 0.28 fSNS but with non-significance (*P* values > 0.05) (Supplemental Table 4). For PTN, the positive alleles of *QPTN.uia-6A* showed significant (*P* < 0.001) more tillers per square meter at 9.38 than the negative allele while the two alleles of the *QPTN.uia-4A* did not show a significant difference of PTN (Supplemental Table 4).

## Discussion

### Unique mapping population

The present study used a DH population derived from two high-yielding cultivars that were not segregating for major phenology genes such as *RHT1*, *RHT2*, and *PPD-D1*. No lodging nor significant variations in heading dates among progeny were observed in any of the trials. Using this population facilitated trait assessment and enhanced QTL identification, presumably by reducing effects of genetic background and environment as shown by the high heritability of all traits evaluated and the consistency of the QTL identification in multiple environments.

### Reliability of QTL detection

Although a relatively small population was used for mapping in the present study, the statistical analysis and other supporting validation results showed high reliability of the identified QTL. Detection power (Rosner 2010) was extremely high from 0.94 to 1 (the maximum power value is 1) for most of the identified QTL and moderately high at 0.80 for *QfSNS.uia-7A* (Table 3), indicating highly reliable QTL identification. In addition, the highly correlated linear relationship between the genetic map and the 90K consensus map as well as the physical map of chromosomes 4A, 5A, 6A, and 7A indicated the high reliability of our linkage map construction. Finally, the allelic analyses of the selected homozygous RILs validated all QTL effects identified in the DH population. All these results suggest that the QTL identification in the present study was valid and reliable.

### Comparative study of the QTL identified for PTN and fSNS

QTL for PTN and fSNS have been studied by others (Richards 1988; Shah et al. 1999; Kato et al. 2000; Li et al. 2002; Huang et al. 2003; An et al. 2006; Narasimhamoorthy et al. 2006; Kumar et al. 2007; Deng et al. 2011). Below are comparisons of QTL identified in the present study with those previously described.

#### Chromosomes 4A and 7A

In the present study, a QTL pair on the long arm of chromosome 4A was identified for PTN (*QPTN.uia-4A*) and fSNS (*QfSNS.uia-4A*) and one QTL was identified at the end of long arm of chromosome 7A for fSNS (*QfSNS.uia-7A*). Jantasuriyarat et al. (2004) identified three QTL for spikelet number per spike on the long arms of chromosomes 4A and 7A. However, because RFLP markers were used in that study, no precise comparison can be made with our results. In addition, Zhang et al. (2015), Quarrie et al. (2005), and Luo et al. (2016) each identified one QTL, including *TaMOC1*, for spikelet number on the long arm of chromosome 7A. However, based on the physical positions, these QTL are closer to the centromere region rather than the telomere region and thus differ from the one described here. Recently, Zhang et al. (2018) identified a SNP marker (*IWA5912*) that was significantly associated with the spikelet number per spike in a genome-wide association study. This marker was located at the 674 Mbp of the chromosome of 7A, a region near where *QfSNS.uia-7A* is located.

In summary, the present work is likely the first to identify a QTL pair on chromosome 4A affecting PTN and fSNS and also validated a QTL for fSNS on the long arm of chromosome 7A.

#### Chromosome 5A

Several genes affecting adaptability and productivity are located at the long arm of chromosome 5A in wheat (Law and Worland 1973; Snape et al. 1985; Miura and Kuroshima 1996), including one of the main determinants of the winter/spring growth-habit polymorphism, vernalization gene *VRN-A1*, and the ear morphology gene *Q*. In the present study, as shown in Fig. 2d, *VRN-A1* was mapped 31 cM from the peak of *QfSNS.uia-5A*, corresponding to 49 Mbp in the physical map, suggesting that *VRN-A1* does not correspond to *QfSNS.uia-5A*. This conclusion was further supported by the allele analysis, which showed no effect of *VRN-A1* on fSNS (Supplemental Fig. 2). In contrast, the *Q* locus is located at the flanking region of *QfSNS.uia-5A*. However, considering the large effect of *Q* on spike morphology and the fact that the spikes of both parents and the DH population appeared normal, it is unlikely the *Q* is the determent for *QfSNS.uia-5A*.

In the region between *VRN-A1* and *Q*, Kato et al. (2000) identified a QTL for spikelet number, which could be the same QTL identified in the present study. For other regions on the long arm of chromosome 5A, previous studies identified a few QTL for yield component traits. For example, Ma et al. (2007) detected a QTL for spikelet number between the markers *RAC875_C1503_642* and *wsnp_Ex_ c20352_29416468*. Gadaleta et al. (2014) identified candidate genes for yield components in the region ranging from *IWB47196* to *IWB35454*. Both QTL regions are located at the middle of the long arm of chromosome 5A and are more than 100 Mbp away from the QTL identified in the present study.

#### Chromosome 6A

*QfSNS.uia-6A* and *QPTN.uia-6A* were mapped to the proximal region of the short arm of chromosome 6A. Previous studies have reported QTL for tiller number on chromosomes 6A, 6B, and 6D (Li et al. 2002; Huang et al. 2003; An et al. 2006; Kumar et al. 2007; Naruoka et al. 2011). An et al. (2006) identified a QTL for tiller number on chromosome 6A flanked by wmc179 and wmc256 and Naruoka et al. (2011) identified a QTL for PTN on the same chromosome flanked by gpw4312 and gpw4145. However, the QTL detected in those studies were located on the long arm of chromosome 6A based on the Chinese Spring deletion map, and thus differ from the 6A QTL identified in the present study. Consequently, the QTL on chromosome 6A found in the present study are likely novel.

### QTL effects in the diverse spring wheat panel

Given the different QTL reported by previous studies, it is likely that many of the identified QTL for fSNS and PTN maybe population (genetic background) specific. In this study, we genotyped a diverse spring wheat panel using the KASP markers and conducted the allelic analysis based on 2 years’ phenotyping data of PTN and fSNS. As the result showed, *QPTN.uia-4A*, *QfSNS.uia-4A*, and *QfSNS.uia-5A* had no effect on these two traits in this panel, indicating they maybe population-specific QTL. On the other hand, positive alleles of *QPTN.uia-6A*, *QfSNS.uia-6A*, and *QfSNS.uia-7A* significantly increased PTN/fSNS in the diverse panel, suggesting they are more universal in their effects. These results can help us and other breeders to make decisions on which QTL will be pyramided in a specific germplasm.

### Challenges for selecting desirable combinations for PTN and fSNS

The genetic architecture and regulating network for spike-related traits are complicated. It is well known that plants can balance spike number, spikelet number, kernel number, and kernel weight in response to environmental variation (Griffiths et al. 2015; Quintero et al. 2014). However, the knowledge of the molecular mechanisms underlying these regulations is limited. In the present study, we found a trade-off relationship between PTN and fSNS based on the phenotypic correlation. Then, we identified two QTL pairs on chromosomes 4A and 6A that affected both PTN and fSNS. Further allelic analysis indicated that the SYC allele of the two QTL pairs increased PTN but decreased fSNS, suggesting that high productive tiller number allele of these QTL pairs decreases the fSNS. This explains at the molecular level why selection of both high PTN and fSNS has been exceptionally difficult with conventional breeding.

### Relationship between PTN and fSNS with thousand kernel weight and yield in the DH population

Yield is a complex trait and contributed by three major yield component traits, including tiller number per area, kernel number per spike, and kernel weight. In the case of the DH population used in this study, using the phenotyping data collected in the same trials described in this study, correlations between PTN and fSNS with thousand kernel weight (TKW) and yield (YLD) were not significant (all *P* values > 0.05 and data not shown), indicating there are no direct relationships between PTN and fSNS with TKW and YLD. Furthermore, using the same genotyping data, the QTL for TKW were identified on chromosomes 2D, 3D, and 5D, while the QTL for YLD were identified on chromosomes 2A and 6B (Liu et al. 2018b). No common QTL were identified between PTN and fSNS with TKW and YLD. Also, the positive and negative alleles of all the six QTL for PTN or fSNS make no difference on TKW and YLD (all *P* values > 0.05 and data not shown).

## Conclusion

The present study identified two novel QTL/QTL pairs on chromosomes 4A and 6A and confirmed previously reported QTL on chromosomes 5A and 7A for two important yield components. KASP markers for the four QTL/QTL pairs will be useful in specific/diverse populations towards pyramiding of multiple QTL of yield components and potentially increasing yield. The next step is to dissect the two QTL pairs (*QfSNS.uia-4A* and *QPTN.uia-4A*; *QfSNS.uia-6A* and *QPTN.uia-6A*) and select desirable recombinants in the 600 RILs that were generated.

## Electronic supplementary material

Supplemental Fig. 1 Boxplots showing additive effect of the identified QTL for PTN and fSNS based on the BLUP data for each trait. Analysis of Variance (ANOVA) test and Tukey method for multiple comparison analysis were used for the comparisons among different allele groups. Capital letters indicate significance level at 0.001 and small letters indicate significance level at 0.05. The number in the X-axis indicated the number of positive alleles in that group. (PNG 118 kb)

High resolution image (TIF 559 kb)

Supplemental Fig. 2. Effect of *VRN-A1* gene on fSNS. T-test analyses were used to compare the two different allele groups, ns indicates no significance. (PNG 59 kb)

High resolution image (TIF 255 kb)

ESM 1 (DOCX 27 kb)
