# Supplementary figures and images for: QTL identification and KASP marker development for productive tiller and fertile spikelet numbers in two high-yielding hard white spring wheat cultivars
Source: Mol Breed. 2018 Nov 1;38(11):135. doi: 10.1007/s11032-018-0894-y (PMC6223832; doi:10.1007/s11032-018-0894-y)

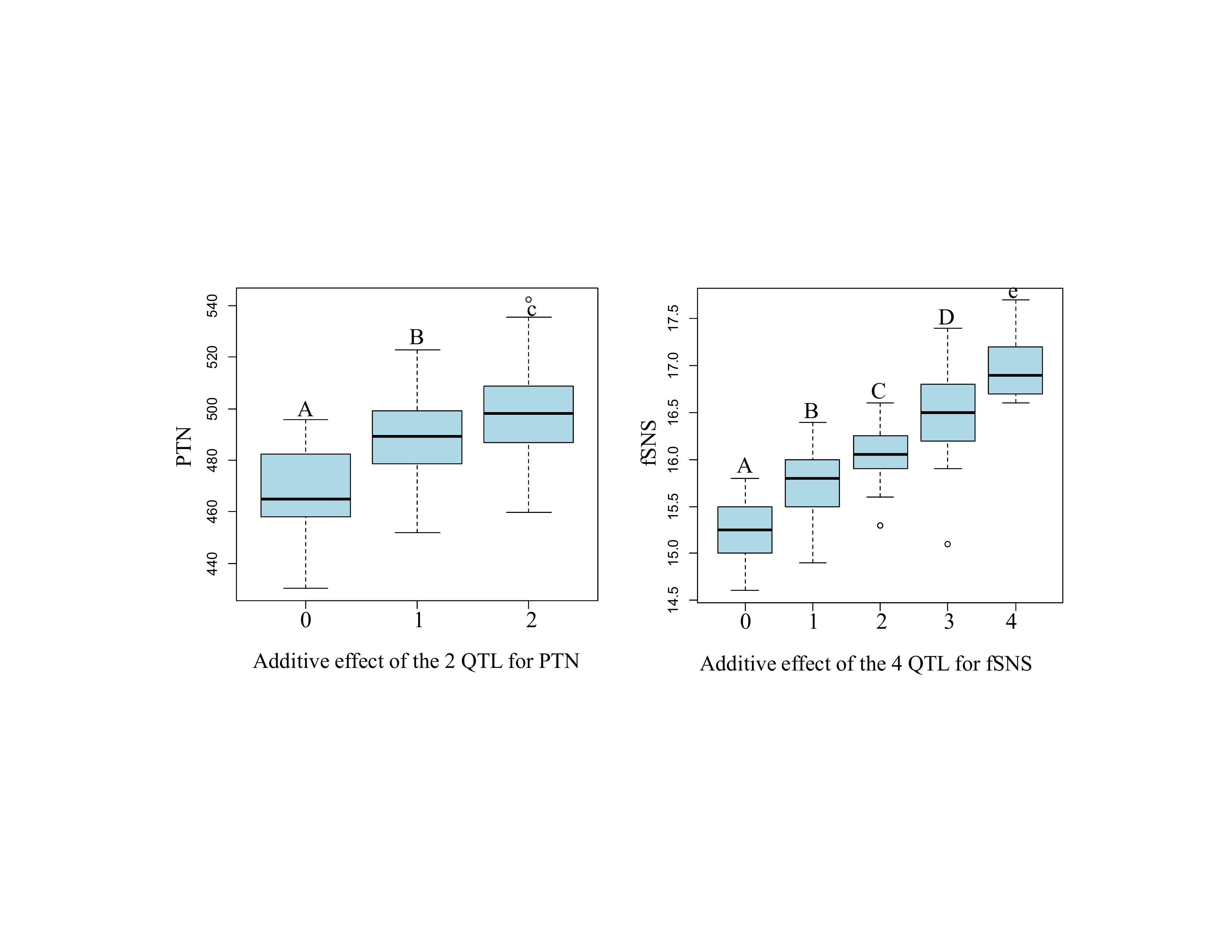

Supplement: Supplementary file 1 — Boxplots showing additive effect of the identified QTL for PTN and fSNS based on the BLUP data for each trait. Analysis of Variance (ANOVA) test and Tukey method for multiple comparison analysis were used for the comparisons among different allele groups. Capital letters indicate significance level at 0.001 and small letters indicate significance level at 0.05. The number in the X-axis indicated the number of positive alleles in that group. (PNG 118 kb) [file 11032_2018_894_Fig3_ESM.png]

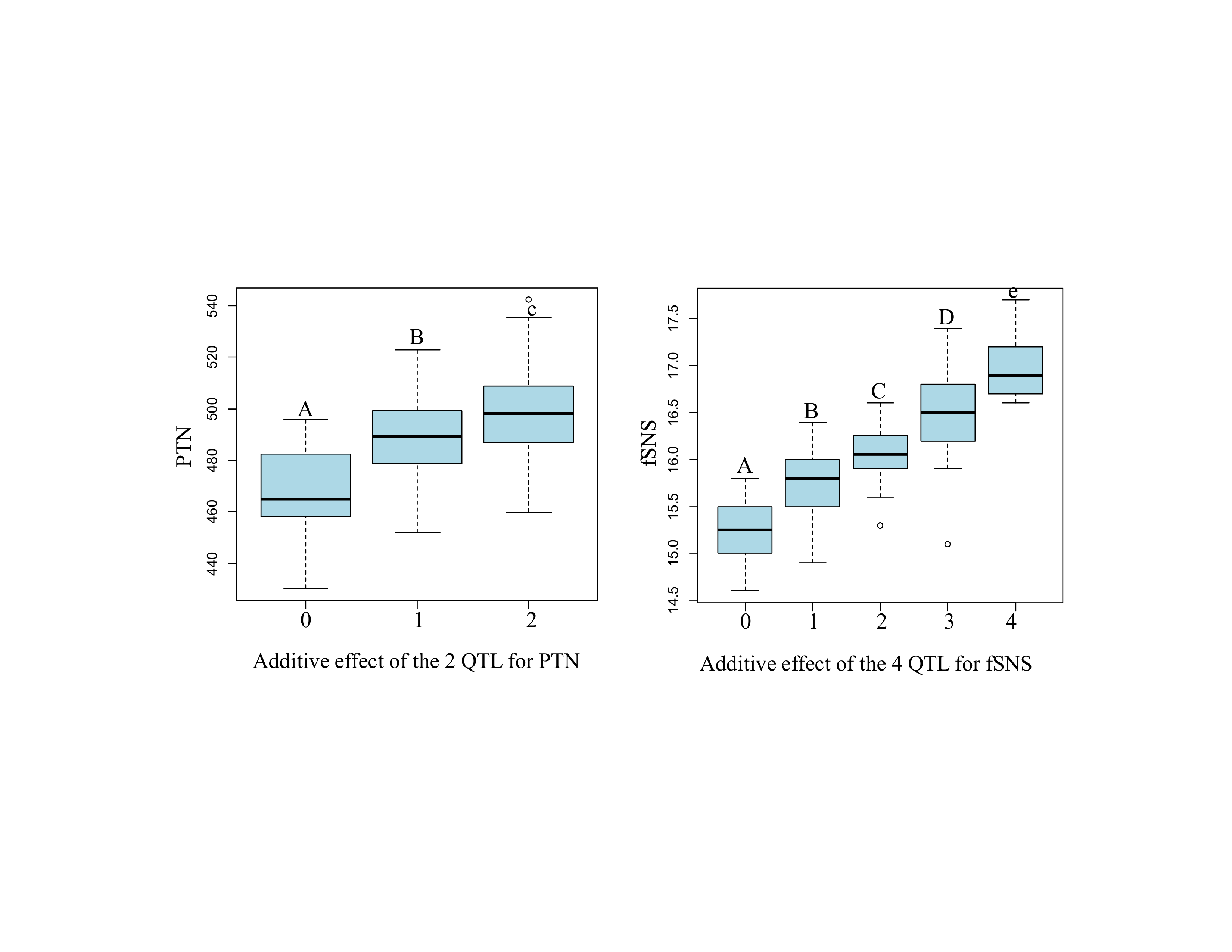

Supplement: Supplementary file 2 — High resolution image (TIF 559 kb) [file 11032_2018_894_MOESM1_ESM.tif]

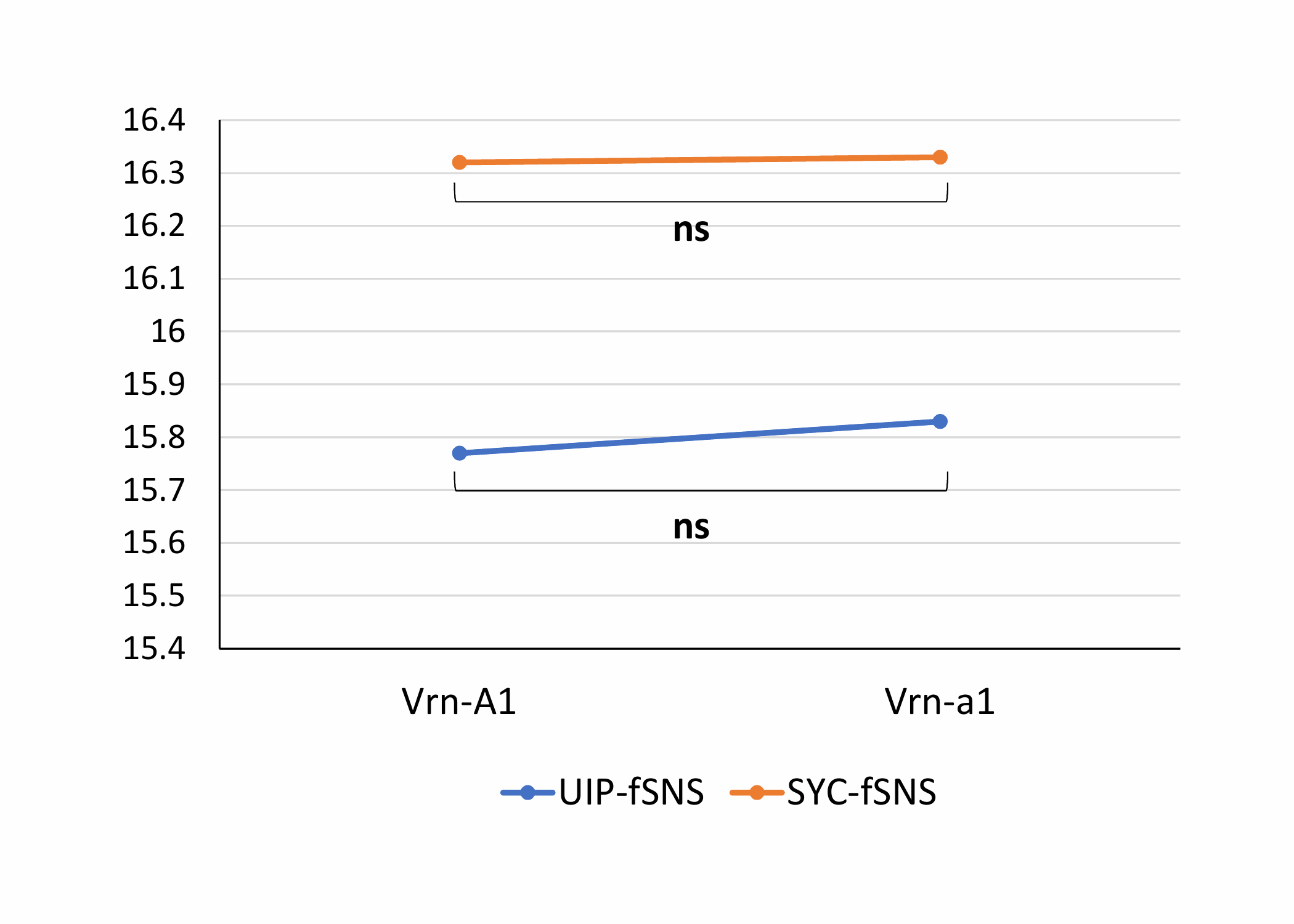

Supplement: Supplementary file 3 — Effect of VRN-A1 gene on fSNS. T-test analyses were used to compare the two different allele groups, ns indicates no significance. (PNG 59 kb) [file 11032_2018_894_Fig4_ESM.png]

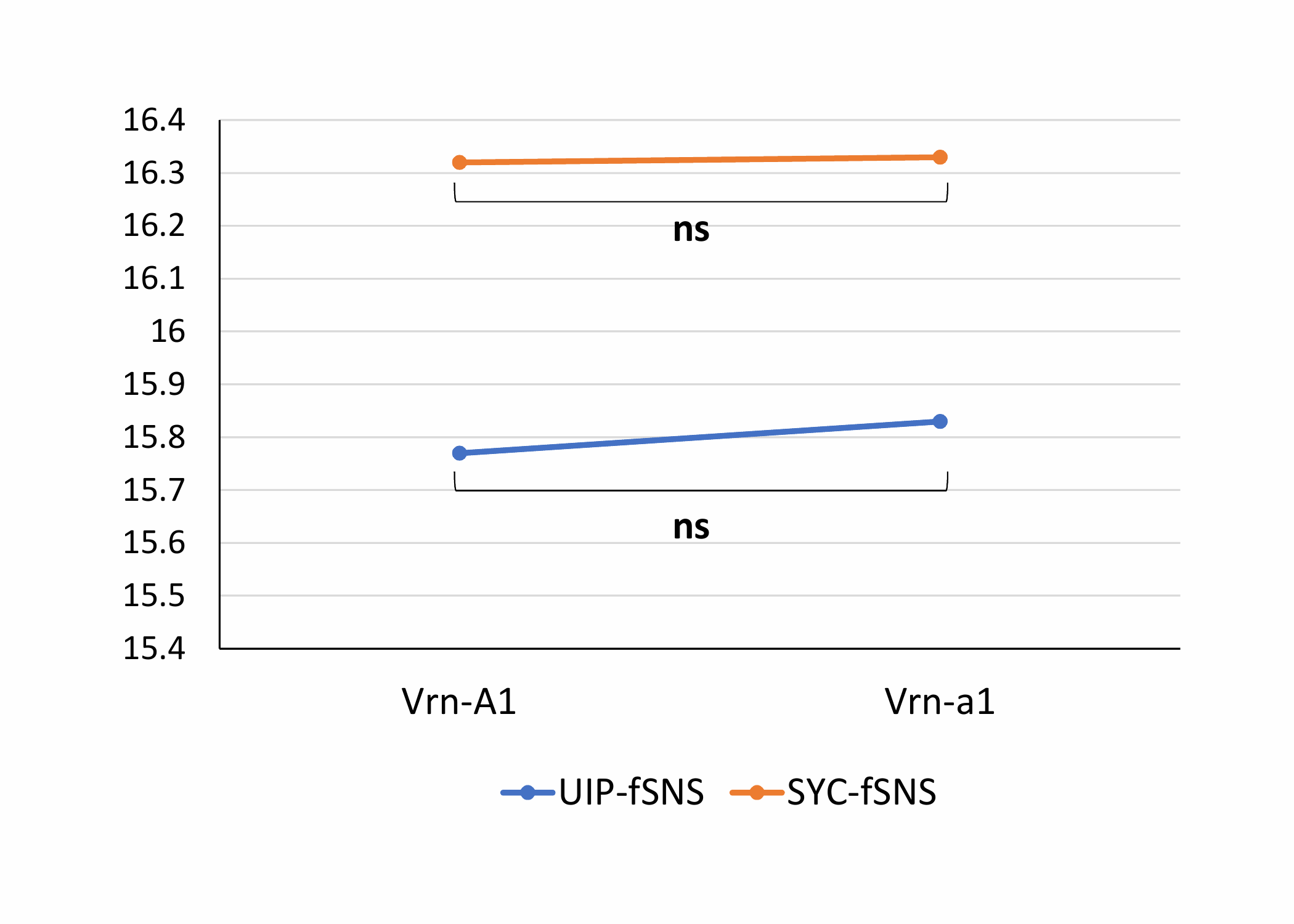

Supplement: Supplementary file 4 — High resolution image (TIF 255 kb) [file 11032_2018_894_MOESM2_ESM.tif]
