# Supplementary material for: QTL identification and KASP marker development for productive tiller and fertile spikelet numbers in two high-yielding hard white spring wheat cultivars
Source: Mol Breed. 2018 Nov 1;38(11):135. doi: 10.1007/s11032-018-0894-y (PMC6223832; doi:10.1007/s11032-018-0894-y)
Supplement: Supplementary file 5 — (DOCX 27 kb) [file 11032_2018_894_MOESM3_ESM.docx]

**Supplemental Table 1. Interactions between QTL for PTN and fSNS using multiple interval mapping method.**

| **PTN** |  |  |  |  |  |
| --- | --- | --- | --- | --- | --- |
| **QTL1** | **QTL2** | **Effect** | ***H0*** | **t Value** | ***P* > \|t\|** |
| *QPTN.uia-4A* | . | Additive2 | Additive2=0 | -4.70 | <.0001 |
| *QPTN.uia-6A* | . | Additive2 | Additive2=0 | -5.21 | <.0001 |
| *QPTN.uia-4A* | QPTN.uia-6A | Additive2 x Additive2 | Interaction=0 | -1.03 | 0.30 |
| **fSNS** |  |  |  |  |  |
| **QTL1** | **QTL2** | **Effect** | ***H0*** | **t Value** | ***P* > \|t\|** |
| *QfSNS.uia-4A* | *.* | Additive2 | Additive2=0 | 7.99 | <.0001 |
| *QfSNS.uia-5A* | *.* | Additive2 | Additive2=0 | -7.78 | <.0001 |
| *QfSNS.uia-6A* | *.* | Additive2 | Additive2=0 | 4.66 | <.0001 |
| *QfSNS.uia-7A* | *.* | Additive2 | Additive2=0 | 6.02 | <.0001 |
| *QfSNS.uia-4A* | *QfSNS.uia-7A* | Additive2 x Additive2 | Interaction=0 | -1.45 | 0.15 |
| *QfSNS.uia-4A* | *QfSNS.uia-5A* | Additive2 x Additive2 | Interaction=0 | -0.83 | 0.41 |
| *QfSNS.uia-5A* | *QfSNS.uia-7A* | Additive2 x Additive2 | Interaction=0 | -1.28 | 0.20 |

The BLUP data for each trait was used to estimate the QTL x QTL interaction effects between different QTL.

**Supplemental Table 2. Information of the KASP markers developed in this study.**

| **QTL/QTL pair^a^** | **KASP marker** | **Genetic position (cM)** | **Primer sequence** | | |
| --- | --- | --- | --- | --- | --- |
|  |  |  | **FAX** | **HEM** | **Common** |
| QTL-4A | K-IWB34531 | 35.51 | TCAAGGACTACCGTCAGTTTTGAT | TCAAGGACTACCGTCAGTTTTGAC | ATGTGTGATGCTGGAAAACG |
|  | K-IWB34374 | 36.42 | GCAACCTATCATACCATCGAAGAGA | GCAACCTATCATACCATCGAAGAGG | TCTCACATACTGGCAGGTAAATAC |
|  |  |  |  |  |  |
| QTL-6A | K-IWB8079 | 109.33 | ACGGCTTATCAATAGTACGACAA | ACGGCTTATCAATAGTACGACAG | AGGAAAATGTACCCATGCTTGA |
|  | K-IWB74471 | 110.24 | CGCTTGGACACGCCTCCT | CGCTTGGACACGCCTCCC | GAGGAGAGGAGGAGACGGTG |
|  | K-IWB54778 | 114.78 | GAGAAGGGTGCACGTAACG | GAGAAGGGTGCACGTAACA | CCTCAGCAAGGCGAAAGG |
|  |  |  |  |  |  |
| QTL-7A | K-IWB7435 | 201.00 | CGCCGCCTGCCAACTAGA | CGCCGCCTGCCAACTAGG | GATTCGCGACACAGTTCCAC |
|  |  |  |  |  |  |
| QTL-5A | K-IWB12226 | 185.48 | TGCTAGGGTTTTGTAGCTCCTGAA | TGCTAGGGTTTTGTAGCTCCTGAC | ATGTACACTTTCTAAAGCTGCATCG |
|  | K-IWB40443 | 187.30 | GATGGAGATCATGCTGG | TGCTGATGGAGATCATGCTGA | GATGAGGATCCATCTCCGAAG |
| ^a^ QTL-4A, QTL-5A, QTL-6A, and QTL-7A stands for the four QTL/QTL pairs on the four chromosomes. | | | | |  |

**Supplemental Table 3. Validation of the QTL and their effects using KASP markers and selected RILs.**

| **QTL pair^a^** | **Trait** | **Mean** | **SD** | ***P* value** | **Sample size** |
| --- | --- | --- | --- | --- | --- |
| **QTL-4A** |  |  |  |  |  |
| UIP alleles^b^ | PTN | 8.81^c^ | 1.12 | 0.0007 | 81 |
| SYC alleles^b^ |  | 9.49 | 1.39 |  | 86 |
| UIP alleles | fSNS | 18.6 | 0.67 | < 0.0001 | 251 |
| SYC alleles |  | 17.53 | 0.49 |  | 203 |
| **QTL-5A** |  |  |  |  |  |
| UIP alleles | fSNS | 17.96 | 0.66 | < 0.0001 | 195 |
| SYC alleles |  | 18.7 | 0.67 |  | 239 |
| **QTL-6A** |  |  |  |  |  |
| UIP alleles | PTN | 8.58 | 1.16 | < 0.0001 | 72 |
| SYC alleles |  | 9.49 | 1.37 |  | 89 |
| UIP alleles | fSNS | 18.61 | 0.72 | < 0.0001 | 186 |
| SYC alleles |  | 18.20 | 0.74 |  | 190 |
| **QTL-7A** |  |  |  |  |  |
| UIP alleles | fSNS | 18.50 | 0.76 | 0.0013 | 275 |
| SYC alleles |  | 18.28 | 0.71 |  | 254 |

^a^ QTL-4A, QTL-5A, QTL-6A, and QTL-7A stands for the four QTL/QTL pairs on the four chromosomes.

^b^ UIP or SYC alleles group stands for the lines with the alleles of designed KASP markers for multiple QTL in a specific QTL pair come from UIP or SYC.

^c^ T-test analyses were used to compare the two different allele groups.

**Supplemental Table 4. Effects of the identified QTL in a diverse spring wheat panel using KASP markers**

| **QTL pair^a^** | **Trait** | **Mean** | ***P* value** | **Sample size** |
| --- | --- | --- | --- | --- |
| **QTL-4A** |  |  |  |  |
| UIP alleles^b^ | PTN | 450.34^c^ | > 0.05 | 73 |
| SYC alleles^b^ |  | 452.25 |  | 91 |
| UIP alleles | fSNS | 17.63 | > 0.05 | 73 |
| SYC alleles |  | 17.35 |  | 91 |
| **QTL-5A** |  |  |  |  |
| UIP alleles | fSNS | 17.43 | > 0.05 | 108 |
| SYC alleles |  | 17.56 |  | 57 |
| **QTL-6A** |  |  |  |  |
| UIP alleles | PTN | 457.37 | < 0.001 | 73 |
| SYC alleles |  | 447.99 |  | 88 |
| UIP alleles | fSNS | 17.66 | < 0.01 | 73 |
| SYC alleles |  | 17.04 |  | 88 |
| **QTL-7A** |  |  |  |  |
| UIP alleles | fSNS | 17.80 | < 0.001 | 52 |
| SYC alleles |  | 16.97 |  | 115 |

^a^ QTL-4A, QTL-5A, QTL-6A, and QTL-7A stands for the four QTL/QTL pairs on the four chromosomes.

^b^ UIP or SYC alleles group stands for the lines with the alleles of designed KASP markers for multiple QTL in a specific QTL pair come from UIP or SYC.

^c^ T-test analyses were used to compare the two different allele groups.
